# Area summation is related to efficient neural representation

**DOI:** 10.1186/1471-2202-16-S1-P71

**Published:** 2015-12-18

**Authors:** Fariba Sharifian, Hanna Heikkinen, Ricardo Vigário, Simo Vanni

**Affiliations:** 1 Brain Research Unit, Department of Neuroscience and Biomedical Engineering, Aalto University, Espoo, Finland; 2 AMI Centre, Aalto Neuroimaging, Aalto University, Espoo, Finland; 3 Clinical Neurosciences, Neurology, University of Helsinki, Helsinki, Finland; 4 Helsinki University Hospital, Helsinki, Finland; 5 Department of Information and Computer Science, Aalto University School of Science, Aalto University, Espoo, Finland

## 

In the primary visual cortex (V1), increasing stimulus size first increases and then decreases the neural firing rate before reaching an asymptote [1,2]. This typical response curve, area summation function (ASF), defines canonical contextual modulation in typical V1 neuron. Previous studies suggest that contextual modulation associates with efficiency of the neural network [3,4]. Here, we studied the relationship between ASF and efficiency of system level neural activation patterns.

Cavanaugh et al. [1] provided quantitative data from macaque cortex, as well as a mathematical description for the ASF. Therefore, we created a theoretical expected cortical response for an arbitrary gray-level image input stimulus based on ASF. Next, we used a biophysically meaningful (biomimetic) network of exponential integrate-and-fire neurons to stimulate V1 response (Heikkinen, Sharifian, Vigario and Vanni, unpublished observations). As an outcome, we compared the distance between the biomimetic simulator outputs to the expected ASF function (Distance to Area Summation, DAS). Finally, we studied entropy per spike, energy consumption and neural population sparseness as a function of the DAS for 20 natural grayscale images. In the biomimetic simulation we left V1-extrastriate and V1 excitatory-inhibitory connection strengths as free parameters in the simulations. A square search with 626 combinations of values was run for all the 20 natural stimuli resulting in 20 * 626 = 12520 simulated response patterns.

**Figure 1:**
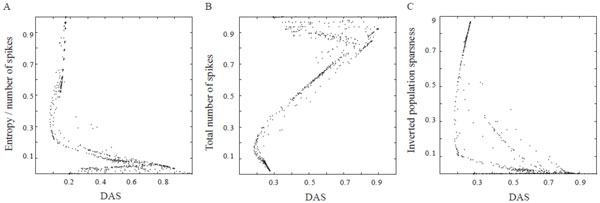


Our results show that: i) there is a clear association between the entropy per spike and DAS (distance correlation = 0.66, Fig 1.a). ii) A negative link exists between the level of energy consumption (linearly associated with spike frequency) in modeled output patterns and DAS (r = 0.62, p < 0.001, Fig 1.b). iii) Population sparseness of the modeled output patterns is positively related to the DAS (r = 0.60, p < 0.001, Fig 1.c).

In conclusion, the results of our study suggest that there is a link between area summation function and efficient information coding in the cortical visual system.
